# MiR-21 is an Ngf-Modulated MicroRNA That Supports Ngf Signaling and Regulates Neuronal Degeneration in PC12 Cells

**DOI:** 10.1007/s12017-014-8292-z

**Published:** 2014-02-04

**Authors:** Enrica Montalban, Nicola Mattugini, Roberta Ciarapica, Claudia Provenzano, Mauro Savino, Fiorella Scagnoli, Gianluca Prosperini, Claudia Carissimi, Valerio Fulci, Carmela Matrone, Pietro Calissano, Sergio Nasi

**Affiliations:** 1 Istituto di Biologia e Patologia Molecolari (IBPM) CNR, Dipartimento di Biologia e Biotecnologie Università Sapienza, 00185 Rome, Italy; 2 European Brain Research Institute (EBRI), Via del Fosso di Fiorano 64, 00143 Rome, Italy; 3 Istituto di Biologia Cellulare e Neurobiologia (IBCN) CNR, Via del Fosso di Fiorano 64, 00143 Rome, Italy; 4 CINECA, Via dei Tizii 2/c, Rome, Italy; 5 Dipartimento di Biotecnologie Cellulari ed Ematologia, Università Sapienza, 00185 Rome, Italy; 6 Present Address: Institut du Fer à Moulin—UMR-S 839—INSERM—UMPC, 17, rue du Fer à Moulin, 75005 Paris, France; 7 Present Address: Department of Physiological Genomics, Institute of Physiology Ludwig-Maximilians Universität (LMU), Schillerstraße, 46, 80336 Munich, Germany; 8 Present Address: Department of Medical Biochemistry, University of Aarhus, Aarhus, Denmark

**Keywords:** Neurotrophins, MicroRNA, Differentiation, Signaling, Neurodegeneration, Gene expression

## Abstract

The neurotrophins Ngf, Bdnf, NT-3, NT4–5 have key roles in development, survival, and plasticity of neuronal cells. Their action involves broad gene expression changes at the level of transcription and translation. MicroRNAs (miRs)—small RNA molecules that control gene expression post-transcriptionally—are increasingly implicated in regulating development and plasticity of neural cells. Using PC12 cells as a model system, we show that Ngf modulates changes in expression of a variety of microRNAs, including miRs known to be modulated by neurotrophins—such as the miR-212/132 cluster—and several others, such as miR-21, miR-29c, miR-30c, miR-93, miR-103, miR-207, miR-691, and miR-709. Pathway analysis indicates that Ngf-modulated miRs may regulate many protein components of signaling pathways involved in neuronal development and disease. In particular, we show that miR-21 enhances neurotrophin signaling and controls neuronal differentiation induced by Ngf. Notably, in a situation mimicking neurodegeneration—differentiated neurons deprived of Ngf—this microRNA is able to preserve the neurite network and to support viability of the neurons. These findings uncover a broad role of microRNAs in regulating neurotrophin signaling and suggest that aberrant expression of one or more Ngf-modulated miRs may be involved in neurodegenerative diseases.

**Electronic supplementary material:**

The online version of this article (doi:10.1007/s12017-014-8292-z) contains supplementary material, which is available to authorized users.

## Introduction

Neurotrophins—nerve growth factor (Ngf), brain-derived neurotrophic factor (Bdnf), neurotrophin 3 (NT3), and neurotrophin 4–5 (NT4–5)—are a family of growth factors that regulate neuronal development, survival, synapse formation, and plasticity (Chao et al. 2006). They act through multiple mechanisms triggered upon binding to two kinds of cell surface receptors: a Trk tyrosine kinase receptor, specific for the single neurotrophins—TrkA, B, C—and the p75 neurotrophin receptor, bound by all of them (Kaplan and Miller 2000). Binding to Trk receptors activates intracellular signaling pathways—including extracellular signal-regulated kinase/mitogen-activated protein kinase (ERK/MAPK), phosphoinositide 3-kinase (Pi3K), and phospholipase C (PLC) pathways—that lead to phosphorylation of transcription factors like Creb and trigger changes in the expression of hundreds mRNAs encoding proteins such as c-Fos, Tyrosine Hydroxylase, Vgf, Bcl-2 (Kaplan and Miller 2000; Annibali and Nasi 2007). Increasing evidence of the implication of miRs in differentiation and plasticity of neurons (Kosik 2006), suggests that transcriptional regulation of miR expression may represent a relevant mechanism mediating neurotrophin functions (Vo et al. 2005; Remenyi et al. 2010; Terasawa et al. 2006). The clearest example is transcriptional regulation of the miR-212/132 cluster by Bdnf via ERK/MAPK signaling and Creb phosphorylation (Vo et al. 2005; Remenyi et al. 2010; Wanet et al. 2012). Mir-212 and miR-132 have roles in a variety of processes including neuronal differentiation, dendritic arborization, synaptic plasticity, and cognitive processes (Tognini et al. 2011; Luikart et al. 2011). Their deregulation is associated with brain-related diseases, such as schizophrenia (Miller and Wahlestedt 2012), bipolar disorder, neurodegenerative diseases (Wanet et al. 2012).

Ngf—the prototype neurotrophin—has a role in peripheral and central nervous system neurons, such as forebrain cholinergic neurons. Ngf signaling defects have been implicated in aging and Alzheimer’s disease (AD) (Calissano et al. 2010; Matrone et al. 2008a, b). To investigate miR expression in response to Ngf, we have employed the PC12 pheochromocytoma cell line, a model for the Ngf action mechanism. PC12 cells respond to Ngf by differentiating into cholinergic neurons and were shown to reproduce certain aspects of the molecular mechanism underlying neurodegeneration in AD (Matrone et al. 2008b). Here, we report the identification of a variety of microRNAs whose expression is modulated by Ngf. We show that the microRNAs have the potential to control several aspects of neuronal development, plasticity, and disease. We report that miR-21—one of the Ngf-modulated miR—regulates Ngf signaling, mediates neuronal differentiation, and prevents neurodegeneration triggered by Ngf removal.

## Results

### Ngf Controls miR Expression in PC12 Cells

To identify microRNAs differentially expressed upon Ngf treatment of naive PC12 cells or upon Ngf deprivation of terminally differentiated cells, we isolated total RNA from PC12 cells: unstimulated, treated with Ngf for 1, 3, 6, 24 h, 10 days, or deprived of Ngf for 24 h after 10 days of treatment with Ngf. We profiled miR expression by a miRCURY LNA™ Array containing over 500 unique miRs and spike-in controls. We normalized microarray expression data with the LOWESS algorithm, and took into account only miRs showing signals significantly higher than background. To compute fold changes, we compared signal intensities of cells treated with Ngf for different times to those of untreated cells. We evaluated differential expression according to the following two criteria: (1) a fold change (FC) of at least 1.7 in absolute value and (2) a *p* value ≤ 0.05. Treatment with Ngf resulted in the upregulation of 68 and downregulation of 44 miRs (Table S1). Raising the FC threshold to two, only 30 miRs appeared to be upregulated and 24 repressed. A small number of microRNAs are modulated at 1–3 h of Ngf treatment: miR-709, -665, -299-3p, -33, -33a, -29c, -130a, miR-691 and few others (Table S1 A, B). The pattern of Ngf-modulated miRs becomes quite complex at 24 h and 10 days of treatment (Table S1 D, E). Early-modulated miRs are expected to be more directly linked to Ngf signaling. Among them, miR-299-3p, miR-709, miR-665, miR-691 were upregulated at early time points—they were no longer upregulated or they were repressed later on—whereas miR-29c, miR-33a, and miR-130a were transiently repressed. MiR-21 and miR-207 were upregulated from 6 h to 10 days (Table S1 C, E). Data are accessible at the Gene Expression Omnibus web site (The Gene Expression Omnibus 2013), DataSet GSE46827.

### Ngf-modulated miRs Affect Neuronal Signaling Pathways

The transcription factor Creb is a key mediator of neurotrophin signaling (Kaplan and Miller 2000; Annibali and Nasi 2007). To assess its involvement in controlling the expression of Ngf-modulated miRs, we investigated the presence of Creb binding sites at their promoters. We employed the JASPAR web site (JASPAR 2013) to interrogate the database of microRNA promoters validated by chromatin immunoprecipitation (Marson et al. 2008). We found a statistically significant enrichment of Creb binding sites at promoters of microRNAs modulated at 3, 6, and 24 h of Ngf treatment, compared with a random promoter population (data not shown). This supports the hypothesis that Ngf may control the expression of early-modulated miRs, at least in part, via Creb.

To get an insight into the possible function of the miRs modulated by Ngf, we made use of DIANA LAB—DNA Intelligent Analysis software (DIANA LAB 2013) to retrieve their predicted targets, and we identified the pathways affected by their expression by means of the KEGG PATHWAY database (KEGG PATHWAY 2013). We retained only pathways with *p* values ≤0.05, calculated by the hypergeometric distribution. Targets of miRs modulated at 1 h were mainly involved in signaling pathways concerning differentiation, proliferation, survival, cell adhesion, TGF-beta signaling, neurotrophin signaling, JAK-STAT signaling, and extracellular matrix (ECM)—receptor interaction (Table 1). Predicted targets of microRNAs modulated by Ngf at 3 and 6 h were preferentially enriched in signaling pathways—MAPK, TGF-beta, p-53 signaling, ECM-receptor interaction—long-term depression, focal adhesion, adherens junction, axon guidance, and glioma. Targets of miRs modulated at 24 h were involved in axon guidance, MAPK signaling, and long-term depression. Targets of miRs modulated at 10 days were strongly enriched in axon guidance, MAPK signaling, and long-term depression pathways. This strongly suggests that several Ngf-modulated miRs may have a role in Ngf signaling.

**Table 1:** MicroRNAs modulated by Ngf appear to be involved in cell signaling

KEGG Pathway	*p* value	#genes	#miRNAs
***A*** *1* *h Ngf*			
TGF-beta signaling pathway	<0.01	15	6
Neurotrophin signaling pathway	<0.01	17	6
Circadian rhythm	0.02	5	5
ECM-receptor interaction	0.02	6	3
SNARE interactions in vesicular transport	0.02	6	6
JAK-STAT signaling pathway	0.02	18	8
***B*** *3* *h Ngf*			
Long-term depression	0.02	9	5
MAPK signaling pathway	0.02	24	5
Focal adhesion	0.04	19	5
TGF-beta signaling pathway	0.04	11	4
***C*** *6* *h Ngf*			
ECM-receptor interaction	<0.01	9	4
Focal adhesion	<0.01	24	7
Axon guidance	0.02	14	6
TGF-beta signaling pathway	0.04	10	6
Adherens junction	0.04	9	6
***D*** *24* *h Ngf*			
Axon guidance	<0.01	30	8
MAPK signaling pathway	0.04	43	7
Long-term depression	0.04	13	6
***E*** *240* *h Ngf*			
TGF-beta signaling pathway	<0.01	50	39
Wnt signaling pathway	<0.01	89	41
Axon guidance	<0.01	74	41
Neurotrophin signaling pathway	<0.01	72	44
Focal adhesion	<0.01	103	40
Long-term potentiation	<0.01	42	40
ErbB signaling pathway	<0.01	48	39
MAPK signaling pathway	0.02	125	43

Predicted targets of all microRNAs modulated by Ngf at each time point—from 1 to 240 h—were retrieved and mapped into the KEGG Pathway database through DIANA miR-Path software; only pathways with *p* value ≤ 0.05 were retained. Pathways relevant to nervous system and neurons are shown; the number of Ngf-modulated miRs targeting a given pathway and the numbers of genes that are targeted in that pathway are also indicated

### Validation of Array Data by Real-Time PCR

**Fig. 1:**
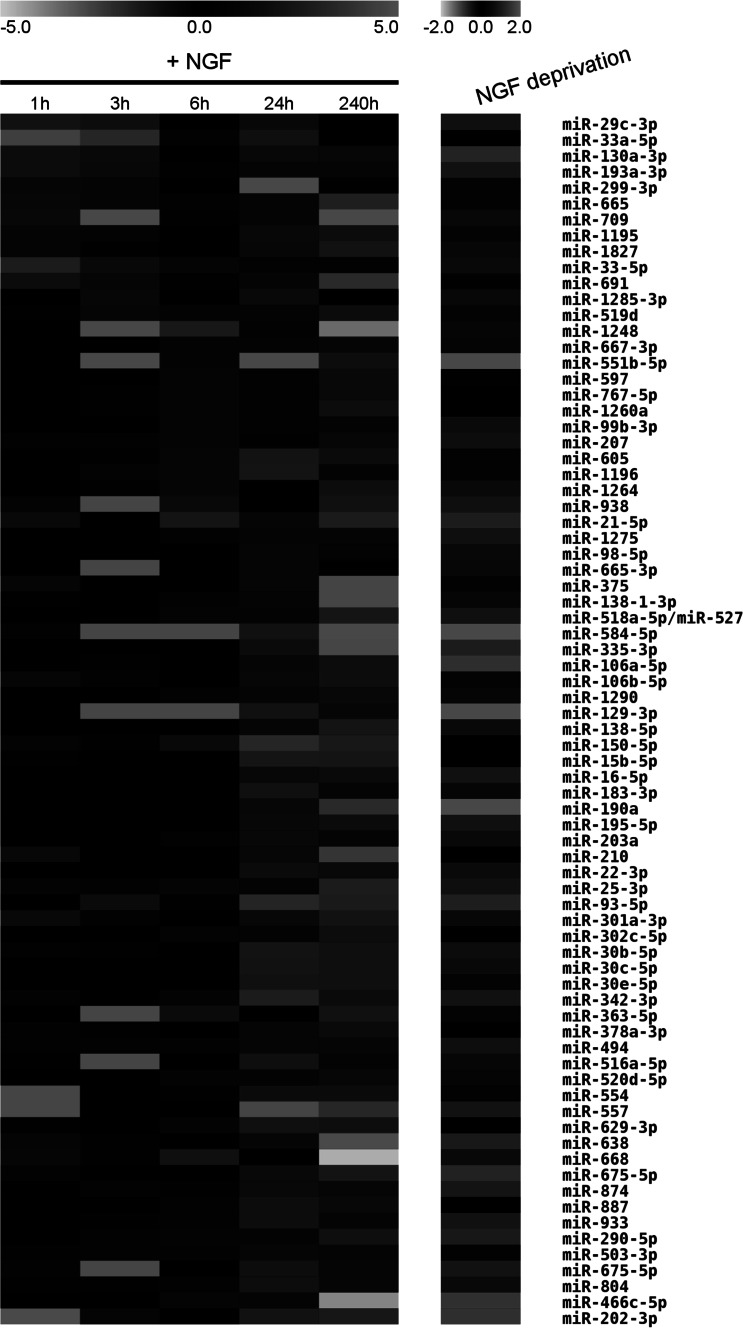
MicroRNAs modulated by Ngf in PC12 cells. Expression values of microRNAs modulated by Ngf are represented as heat map. The *left image* (+Ngf) shows microRNAs differentially expressed in PC12 cells at 1, 3, 6, 24 h and 10 d of Ngf treatment, relative to cells untreated with Ngf. The image on the *right* (Ngf deprivation) refers to microRNA expression in PC12 cells that were treated with Ngf for 10 days and then deprived of Ngf for 24 h, relative to cells treated with Ngf for 10 days but not deprived of Ngf. Only microRNAs modulated by Ngf in at least one condition are shown. *Red* and *green* indicate, respectively, increased or decreased expression relative to controls; *black* indicates no expression change; *gray* indicates that microRNA expression was undetected. Only miR expression values satisfying the threshold criteria—*p* value ≤0.05 in two-tailed Student’s *t* test; fold change ≤−1.7 or ≥1.7—are reported

**Fig. 2:**
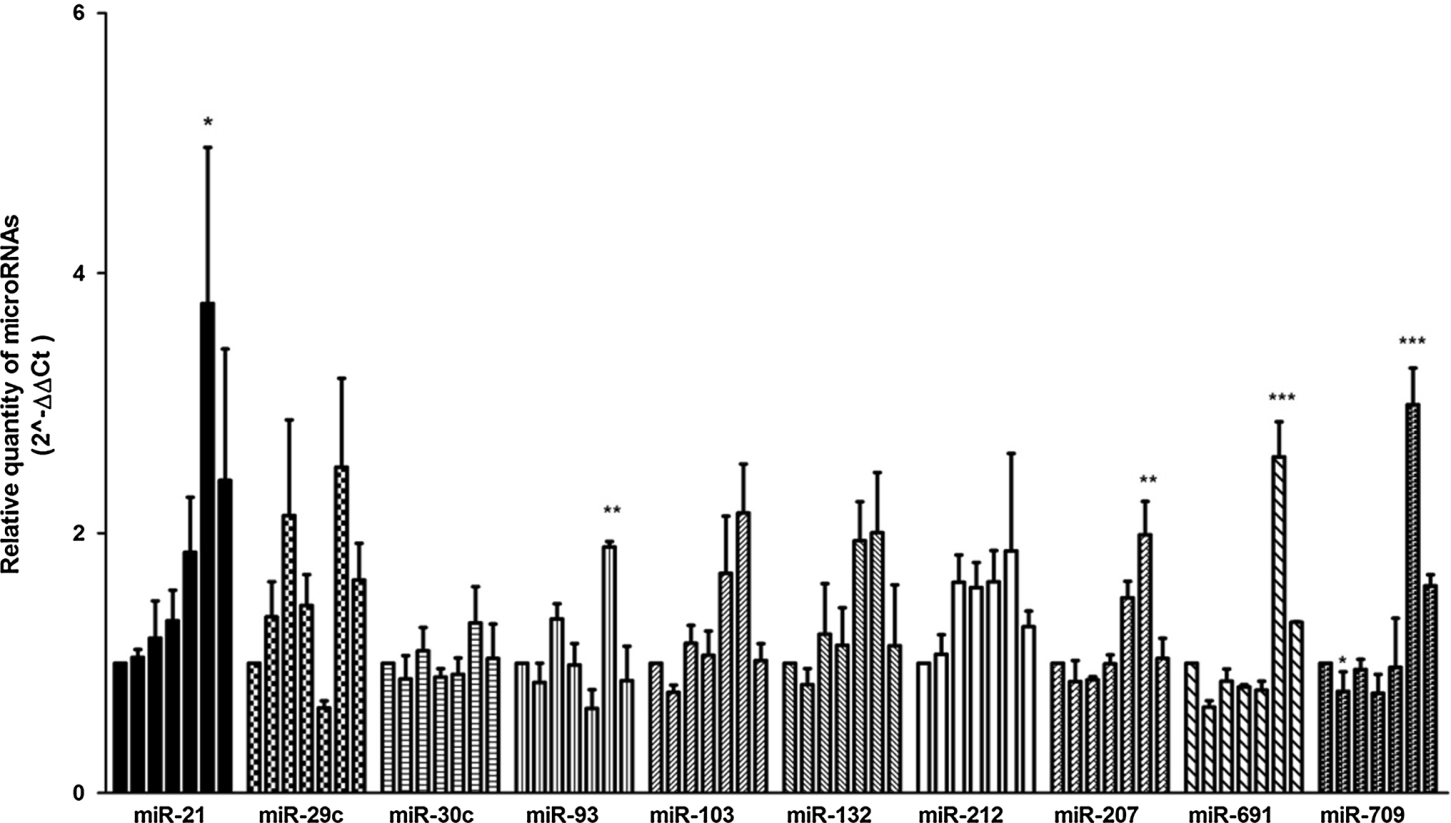
Expression analysis of select microRNAs upon Ngf induction. **a** The plots show the amounts of each of the ten select miRs (21, 29c, 30c, 93, 103, 132, 212, 207, 691, 709) determined by RT-PCR in PC12 cells treated with Ngf—from *left* to *right*, respectively—for 0, 1, 3, 6, 24, 240 h; the most rightward bar of each plot represents cells treated with Ngf for 10 days and deprived of Ngf for 24 h. Expression levels are relative to cells not treated with Ngf. MiR expression values were calculated by the comparative ∆∆Ct method; U6 small nucleolar RNA (snoRNA) was used as internal control. *Data* represent averages of three independent experiments along with s.e.m. *P* values were calculated by one-way ANOVA followed by Tukey’s multiple comparison test; *p < 0.05; **p < 0.03; ***p < 0.01

To validate by quantitative real-time PCR (RT-PCR) miR differential expression upon Ngf treatment, we focused on a set of ten microRNAs, chosen for expression pattern, experimentally proven targets, and literature data. Seven of them—miR-21, miR-29c, miR-30c, miR-93, miR-207, miR-691, miR-709—were picked from the list in Fig. 1. The remaining three microRNAs—miR-103, miR-212, and miR-132—are not listed in Fig. 1 since they presented a subthreshold modulation by Ngf in the array experiments (data not shown), but were selected for the following reasons. Expression of the miR-212/132 cluster in cortical and hippocampal neurons is induced by Bdnf through Creb and their promoter is bound by Creb in PC12 cells (Vo et al. 2005; Remenyi et al. 2010). The two microRNAs are implicated in synaptic plasticity, mental diseases, and drug dependence (Impey et al. 2010; Im et al. 2010). The deregulation of the miR-212/132 cluster correlates with neurological disorders such as AD and tauopathies (Wanet et al. 2012). MiR-103 is induced during differentiation of neuroblastoma cells (Annibali et al. 2012), has several targets in neurotrophin signaling and MAPK pathways, and has been implicated in AD (Yao et al. 2010). We compared RNA samples extracted from untreated cells with samples extracted from PC12 cells stimulated with Ngf for the same periods as in the array experiments—1, 3, 6, 24 h and 10 days of treatment—as well as from cells deprived of Ngf for 24 h after 10 days of treatment with Ngf (Fig. 2). We found a significant correlation between microarray and RT-PCR data as regards differential expression of the seven miRs picked from the list of Ngf-modulated microRNAs. MiR-212 and miR-132 expression as well was modulated by Ngf, as expected for their responsiveness to Bdnf and Creb (Vo et al. 2005; Remenyi et al. 2010). The amount of miR-212 increased starting at 3 h of Ngf exposure and remained higher than control for all successive time points (6, 24 h, 10 days). After 24 h of Ngf treatment, we observed a significant miR-132 increase, still detectable at 10 days. The increased expression levels of the ten miRs appeared to be sustained by ongoing Ngf signaling, since they all returned to a level comparable to naive cells upon deprivation of Ngf for 24 h (Fig. 2). Among them, miR-21 and miR-29c were less sensitive to deprivation of Ngf. This suggests that Ngf has a transient effect upon expression of all these miRNAs in PC12 cells.

To gain an insight into the possible function of the RT-PCR validated microRNAs, we made use of DIANA microT (http://diana.cslab.ece.ntua.gr/microT/) to retrieve putative targets of the set of ten miRs and map them to pathways as described above (Table 2A). The set of their targets was significantly enriched in neurotrophin, MAPK, Akt, mTOR, and TGF-beta signaling pathways as well as in ECM-receptor interaction, axon guidance, focal adhesion, gap junction, and long-term depression, in accordance with Table 1 data.

**Table 2:** Select Ngf-modulated microRNAs appear to affect relevant neuronal pathways

KEGG Pathway	*p* value	#genes	#miRNAs
*A*			
ECM-receptor interaction	9.8e−23	22	7
Axon guidance	8.6e−08	45	9
Focal adhesion	8.6e−08	60	10
Neurotrophin signaling pathway	2.6e−04	37	10
MAPK signaling pathway	5.1e−05	62	10
Gap junction	1.1e−03	20	8
Cytokine–cytokine receptor interaction	1.6e−03	45	10
ErbB signaling pathway	1.9e−03	28	10
mTOR signaling pathway	7.1e−03	17	7
TGF-beta signaling pathway	7.1e−03	23	7
Long-term depression	8.4e−03	16	7
Circadian rhythm	9.0e−03	8	6
Regulation of actin cytoskeleton	2.5e−02	45	9

KEGG Pathway	REFLIST	Observed	*p* value
*B* *miR*-*21 (112 predicted targets)*			
MAPK signaling pathway	259	16	5.7e−06
Cytokine–cytokine receptor interaction	267	14	1.2e−05
PI3 K-Akt signaling pathway	345	12	9.0e−03
Regulation of actin cytoskeleton	214	9	7.3e−03
JAK-STAT signaling pathway	158	9	1.1e−03
Neurotrophin signaling pathway	120	9	1.6e−04
*miR*-*29c (325 predicted targets)*			
PI3 K-Akt signaling pathway	345	51	5.5e−13
Focal adhesion	206	38	7.0e−13
ECM-receptor interaction	87	25	2.5e−13
MAPK signaling pathway	259	18	0.04
Regulation of actin cytoskeleton	214	17	0.02
Neurotrophin signaling pathway	120	15	5.6e−0
Axon guidance	131	14	1.5e−04
Dopaminergic synapse	130	12	0.01
*miR*-*103 (203 predicted targets)*			
PI3 K-Akt signaling pathway	345	17	0.02
MAPK signaling pathway	259	14	0.02
Regulation of actin cytoskeleton	214	12	0.02
Dopaminergic synapse	130	12	5.0e−04
Wnt signaling pathway	143	11	3.2e−03
Neurotrophin signaling pathway	120	7	0.04
*miR*-*30c (424 predicted targets)*			
PI3 K-Akt signaling pathway	345	28	0.04
MAPK signaling pathway	259	26	7.4e−03
Regulation of actin cytoskeleton	214	25	1.5e−03
Axon guidance	131	23	6.7e−06
Focal adhesion	206	19	0.03
Neurotrophin signaling pathway	120	19	1.6e−04
Wnt signaling pathway	143	16	0.01
Dopaminergic synapse	130	16	5.3e−03
Long-term potentiation	71	14	1.1e−04
*miR*-*93 (245 predicted targets)*			
PI3 K-Akt signaling pathway	345	26	3.0e−04
MAPK signaling pathway	259	25	7.7e−06
Regulation of actin cytoskeleton	214	19	2.5e−04
Neurotrophin signaling pathway	120	17	1.6e−06
Axon guidance	131	14	2.6e−04
Focal adhesion	206	14	0.01
Wnt signaling pathway	143	13	1.7e−03
mTOR signaling pathway	60	10	5.5e−05
TGF-beta signaling pathway	81	10	6.3e−04
*miR*-*132 (157 predicted targets)*			
PI3 K-Akt signaling pathway	345	20	1.3e−04
MAPK signaling pathway	259	13	5.6e−03
Axon guidance	131	13	1.1e−05
Regulation of actin cytoskeleton	214	12	3.4e−03
Focal adhesion	206	11	6.7e−03
TGF-beta signaling pathway	81	10	1.8e−05
Dopaminergic synapse	130	9	2.8e−03
Glutamatergic synapse	170	9	0.01
Neurotrophin signaling pathway	120	9	1.7e−03
*miR*-*212 (72 predicted targets)*			
Axon guidance	131	4	0.04
Long-term potentiation	71	3	0.03
*miR*-*691 (177 predicted targets)*			
PI3 K-Akt signaling pathway	345	16	0.01
MAPK signaling pathway	259	11	0.04
Axon guidance	131	11	6.0e−04
Focal adhesion	206	11	0.01
Wnt signaling pathway	143	8	0.02
TGF-beta signaling pathway	81	7	4.6e−03
GABAergic synapse	89	6	0.02
*miR*-*709 (228 predicted targets)*			
PI3 K-Akt signaling pathway	345	21	4.5e−03
Axon guidance	131	16	8.6e−06
Focal adhesion	206	16	1.3e−03
Regulation of actin cytoskeleton	214	14	0.01
ErbB signaling pathway	88	14	1.4e−06
Wnt signaling pathway	143	12	2.7e−03
Dopaminergic synapse	130	12	1.3e−03
Cholinergic synapse	112	9	0.01
Neurotrophin signaling pathway	120	9	0.01
Long-term potentiation	71	7	8.4e−03

*A* KEGG Pathways affected by ten select microRNAs. Predicted targets of the ten select miRs—21, 29c, 30c, 93, 103, 132, 212, 207, 691, 709—were collectively retrieved and mapped into the KEGG Pathway database through DIANA miR-Path software. *B* KEGG Pathways affected by each of the ten select miRs. Predicted targets of each miR were retrieved by TargetScan and mapped into the KEGG Pathway database; only pathways with *p* value ≤ 0.05 were retained. Pathways relevant to nervous system and neurons were considered; mir-207 did not show nervous system relevant pathways with statistical significance. The number of Ngf-modulated miRs targeting a given pathway and the numbers of genes targeted in that pathway are indicated

We then retrieved predicted targets of each single microRNA with TargetScan and mapped them to KEGG Pathway (Table 2B). We focused on nervous system related pathways and we considered only pathways with *p* values ≤0.05. We found over representation of a variety of signaling pathways—Pi3K/Akt, MAPK, neurotrophin, and Wnt signaling—synaptic pathways—glutamergic, dopaminergic, cholinergic, GABA-ergic synapses—and pathways of axon guidance, cell adhesion, long-term potentiation, and AD. A single pathway component was frequently hit by different Ngf-modulated miRs. This indicates that Ngf may control multiple miRs to target the same pathway.

### MiR-21 is a Ngf-Modulated RNA Involved in Ngf Signaling and Differentiation

**Fig. 3:**
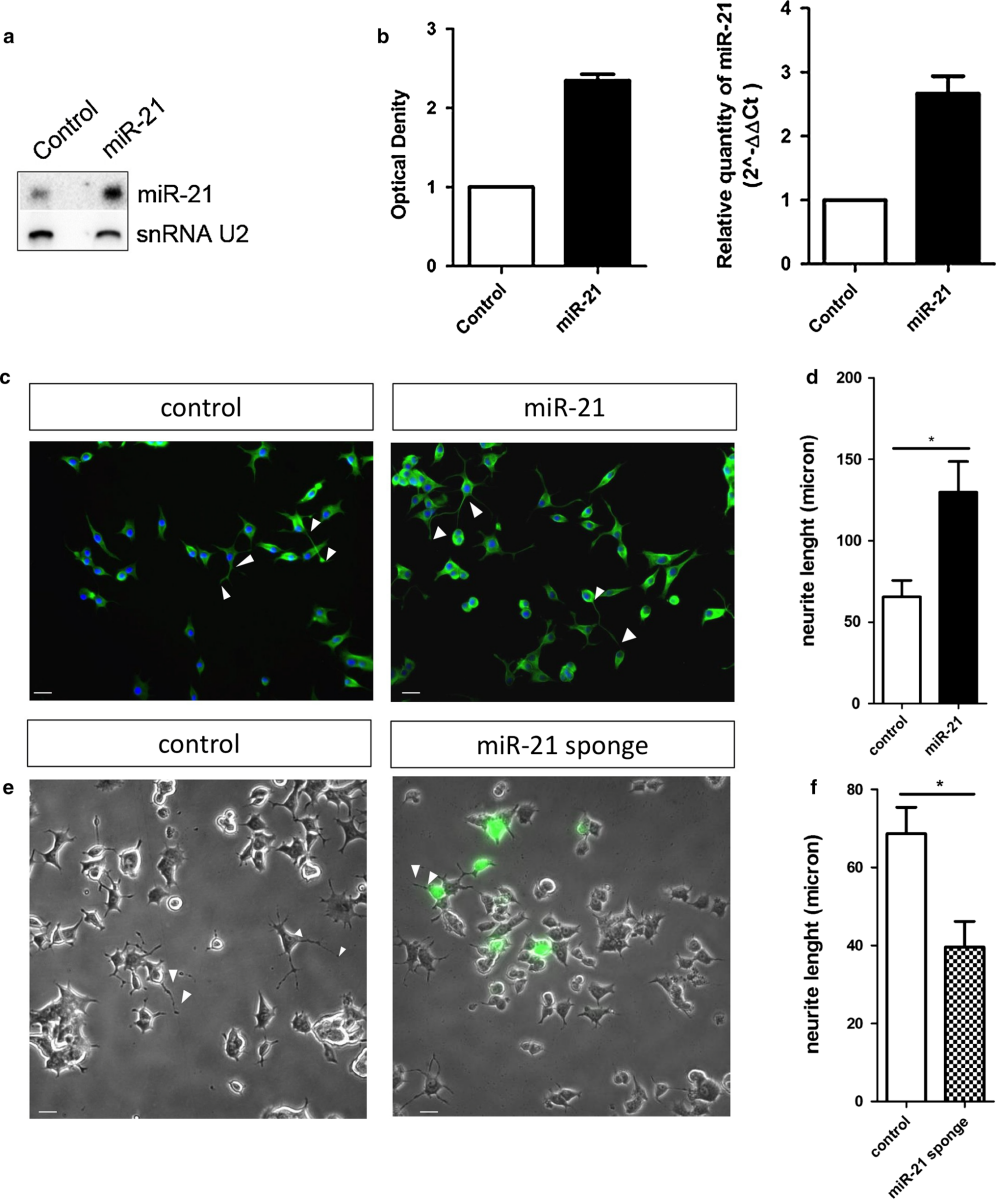
MiR-21 promotes PC12 cell differentiation in response to Ngf. **a**–**b** MiR-21 expression in PC12 cells transduced with miR-21 expressing (miR-21) or control lentivirus. **a** Representative Northern blotting. **b** Histograms showing miR-21 expression—relative to control cells set to a value of 1—measured by Northern blotting (*left*) and real-time PCR (*right*). Northern blotting data are presented as mean ± s.e.m. of two independent experiments; U2 snoRNA was used as loading control. Real-time PCR data are presented as mean ± s.e.m. of triplicate samples, from two biological replicates; U6 snoRNA was used as loading control. **c**–**d** Neurite length of PC12 cells ectopically expressing, or not, miR-21 and stimulated for 2 days with Ngf. **c** Immunofluorescence staining of *α*-tubulin. *Scale bar* 30 μm. **d** Histogram showing the average length of axons, expressed in μm. *Data* are represented as means ± s.e.m. of triplicate determinations of ten random fields in three independent experiments. *P value ≤0.05, according to Student’s t test. **e**–**f** Neurite length of PC12 cells transfected with a miR-21 sponge plasmid—harboring a destabilized EGFP with several miR-21 binding sites in the 3′ untranslated region –, stimulated for 2 days with Ngf, and fixed with 4 % PFA. **e** Photomicrographs. The *green color* represents EGFP; *scale bar* 30 μm. **f** The histogram shows neurite mean length, express in μm; *data* are represented as means ± s.e.m. of triplicate determinations of ten random fields in three independent experiments. *P value ≤0.05, according to Student’s t test

We selected for further investigation miR-21, because it was highly upregulated in PC12 cells from 6 h to 10 days of Ngf treatment and strongly associated to MAPK signaling pathway—a key mediator of Ngf signaling—according to the analysis of Table 2B. These observations suggested a role of this microRNA in Ngf signaling and differentiation. To evaluate this hypothesis, we stably transduced PC12 cells with a miR-21 producing lentivirus (miR-21 cells) and a control lentivirus producing a 22-mer RNA lacking homology to any human gene (control cells). We measured miR-21 expression by Northern blotting and RT-PCR: miR-21 ectopically expressing cells produced 2.8-fold more miR-21 than control cells (Fig. 3a, b). This overexpression level is similar to the one observed in PC12 cells treated with Ngf and can therefore be considered in a physiological range. A 20-fold overexpression of miR-21 was instead toxic to cells (not shown).

**Fig. 4:**
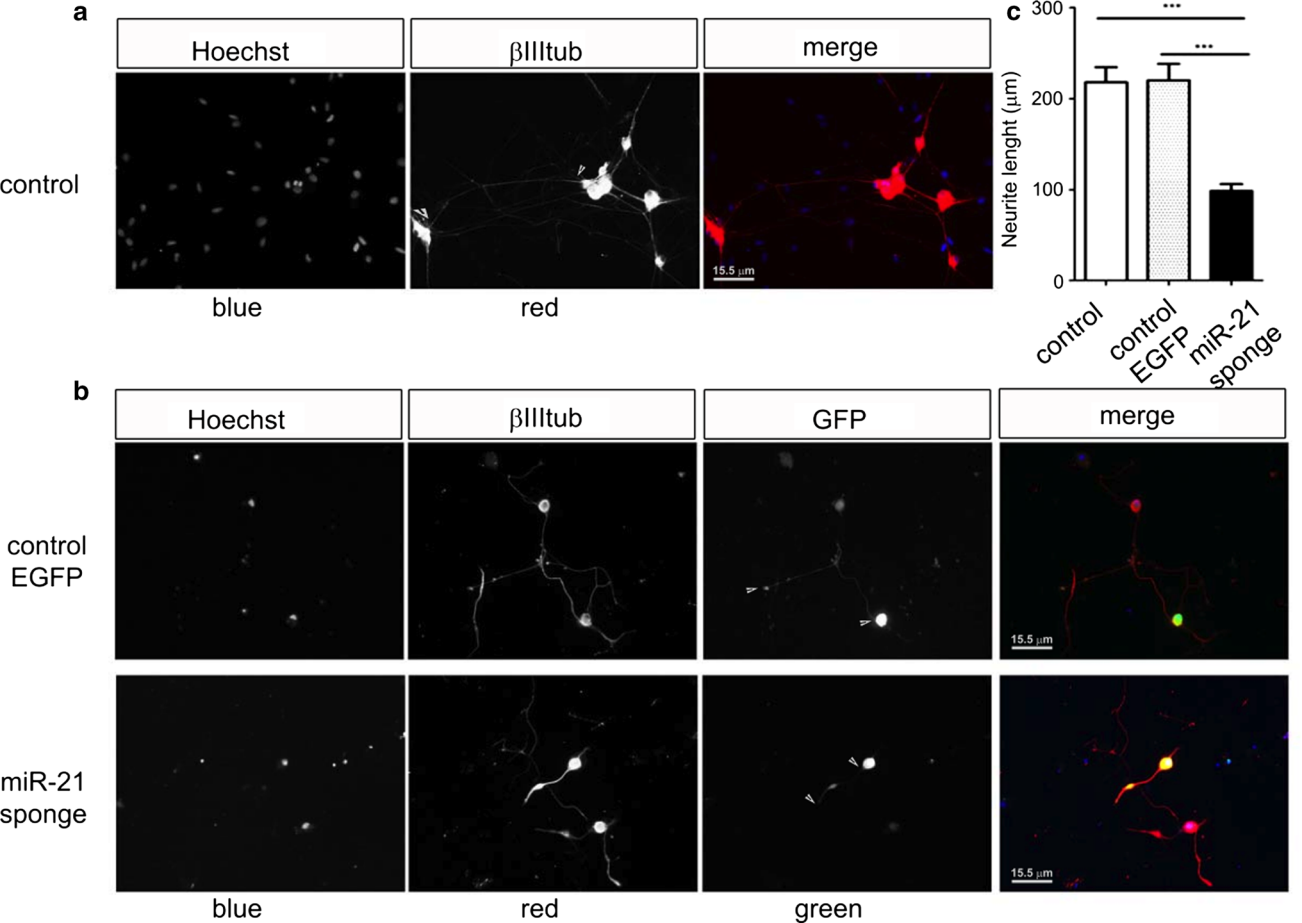
miR-21 inhibition affect neurite outgrowth in DRG cells. **a**, **b** Immunofluorescence staining. **a** Control, untransfected DRG cells, **b** DRG cells transfected with an EGFP encoding plasmid (control EGFP) and with the plasmid encoding the miR-21 sponge. *Blue* Hoechst staining; *red* ß-III-tubulin; *green* GFP. *Scale bar* 15.5 μm. **c** The histogram shows the mean length of neurites express in μm. *Data* are represented as means ± s.e.m. of triplicate determinations of ten random fields in four independent experiments. A total of 169 cells were counted for the not transfected control, a total of 133 cells were counted for EGFP control, and a total of 170 cells were counted for the miR-21 sponge. *P* values were calculated by one-way ANOVA followed by Tukey’s multiple comparison test; ***P value ≤0.01

To determine whether miR-21 ectopic expression affected PC12 cell capacity to undergo neuronal differentiation in response to Ngf, we treated control and miR-21 cells for 2 days with Ngf. We evaluated the length of the main developing axons and found that they were on average longer in cells ectopically expressing miR-21, compared with control cells (Fig. 3c, d). To further support the role of miR-21 in differentiation, we investigated the effect of its inhibition. We transfected PC12 cells with a plasmid expressing a miR-21 sponge (Ebert et al. 2007) and measured the length of developing axons. Figure 3e, f shows a significant decrease of axonal length in cells expressing the miR-21 sponge. To assess whether miR-21 may have similar properties in primary neurons, we repeated the experiment in dorsal root ganglia (DRG) neurons, a highly Ngf responsive population. Dissociated DRGs were transfected with the plasmid encoding a miR-21 sponge and stimulated with Ngf for 12 h. The results show a statistically relevant decrease of axon length in cells transfected with the miR-21 sponge compared to non-transfected cells or to cells transfected with a plasmid encoding an EGFP reporter only (Fig. 4a–c). Consistently with this finding, miR-21 overexpression appeared to increase neurite size in DRG neurons (Figure S1).

Ngf treatment of PC12 cells triggers phosphorylation of MAPK and Akt—a key component of Pi3K signaling—kinases, which remain phosphorylated and active for 1–3 h. To determine whether miR-21 ectopic expression affected Ngf signaling, we measured MAPK and Akt phosphorylation in PC12 miR-21 and control cells upon Ngf treatment for 0 min, 10 min, and 3 h. As shown in the left panel of Figure S2, Akt and MAPK phosphorylation in cells ectopically expressing miR-21 was enhanced at both 10 min and 3 h of Ngf treatment, compared with controls.

**Fig. 5:**
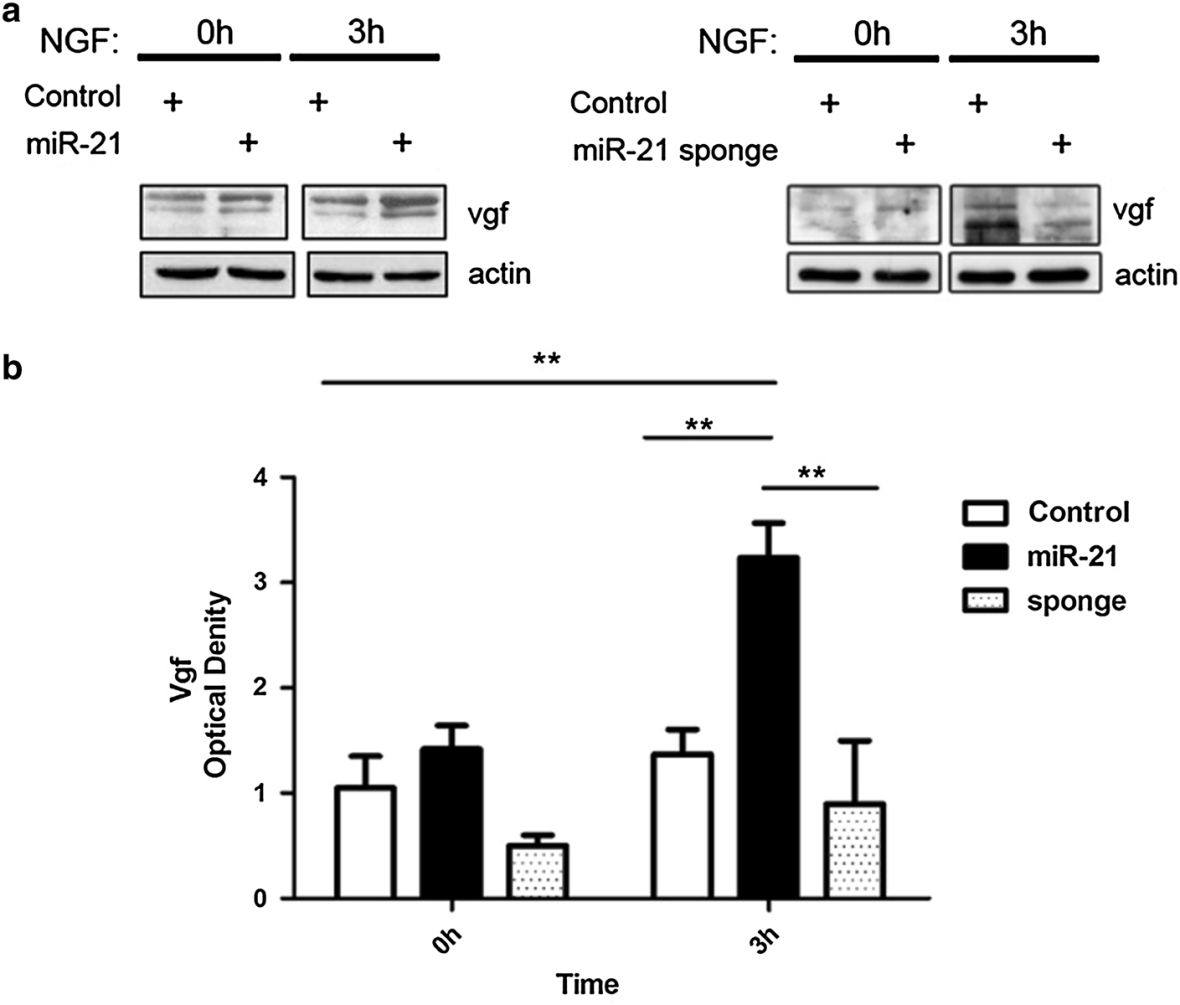
Vgf induction in PC12 cells is affected by miR-21 expression. **a** Vgf immunoblotting in control PC12 cells and cells ectopically expressing miR-21 (*left*) or a miR-21 sponge (*right*), treated with Ngf for 0 and 3 h. **b** Densitometric analyses of the immunoblots—normalized to the actin loading control—of three independent experiments. *Data* are presented as mean ± s.e.m. *P* values were calculated by one-way ANOVA followed by Tukey’s multiple comparison test; **p value ≤0.01

Persistent MAPK activation and Creb transcription factor phosphorylation caused by Ngf and Bdnf signaling mediate the expression of neurospecific genes, such as *vgf*—which encodes a neuropeptide precursor protein. Vgf expression in PC12 cells represents a differentiation marker strictly dependent on Ngf signaling. Activation of *vgf* transcription occurs after 1–3 h and involves Creb binding to a CRE (cAMP response element) in the promoter region (Levi et al. 2004; Mandolesi et al. 2002). To determine whether ectopic expression of miR-21 affected Vgf expression, we treated with Ngf control PC12 cells, cells ectopically expressing miR-21, and cells where miR-21 is inhibited by a lentivirus encoding a miR-21 sponge, and performed immunoblots with Vgf antibodies (Fig. 5). Cells ectopically expressing miR-21 had a higher Vgf level than control cells at 3 h of Ngf treatment (Fig. 5a, left panel). Cells expressing the mir-21 sponge, instead, showed a decreased level of Vgf (Fig. 5a, right panel). This supports the hypothesis that miR-21 may promote expression of neuronal differentiation markers in PC12 cells by enhancing Ngf signaling.

### MiR-21 Protects PC12 Cells from Degeneration Upon Ngf Withdrawal

**Fig. 6:**
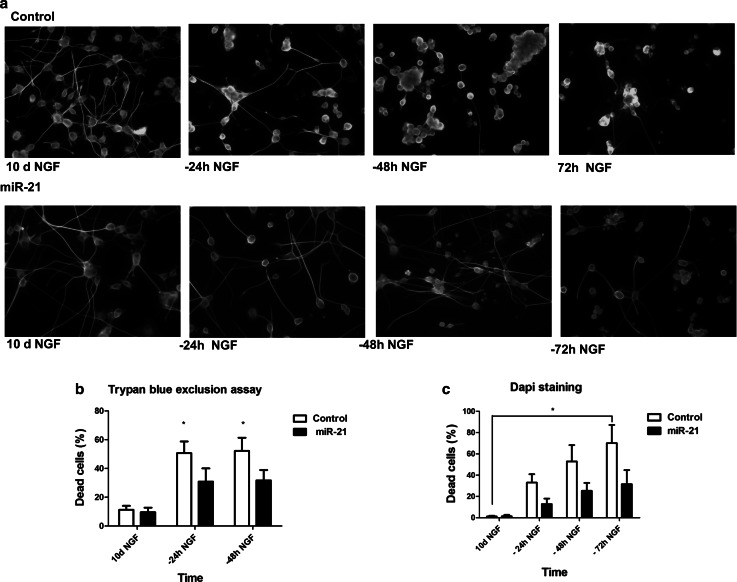
MiR-21 prevents degeneration of differentiated PC12 cells upon deprivation of Ngf. **a** Immunofluorescence staining of *α*-tubulin in PC12 cells, differentiated for 10 days with Ngf (10 d Ngf) and then deprived of Ngf for 24, 48, and 72 h. **b** *Trypan blue* exclusion assay. The histogram indicates percentages of dead cells, at 24 and 48 h after deprivation of Ngf. *Data* are represented as means ± s.e.m. of four independent experiments. **c** Cell viability was evaluated by DAPI staining and counting of intact nuclei. *Data* are represented as means ± s.e.m. of triplicate determinations of ten random fields in three independent experiments. *P* values were calculated by one-way ANOVA followed by Tukey’s multiple comparison test. *P value ≤0.05

**Fig. 7:**
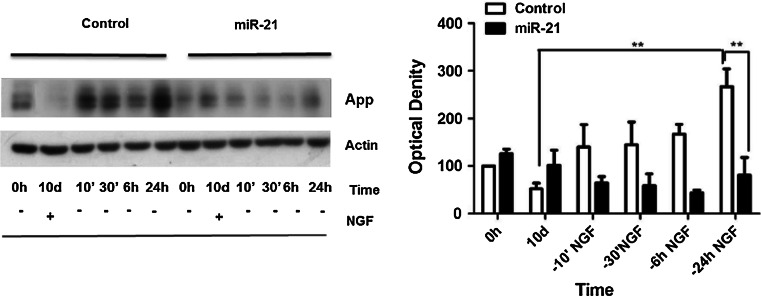
App expression is affected by mir-21 expression. *Left panel* representative immunoblotting of App in PC12 cells ectopically expressing or not miR-21, treated for 10 days with Ngf and then deprived of Ngf from 10 min to 24 h. *Right panel* the histogram depicts densitometric analyses of immunoblots, normalized to the actin loading control. *Data* are represented as means ± s.e.m. of three independent experiments. *P* values were calculated by one-way ANOVA followed by Tukey’s multiple comparison test; **p value ≤0.01 by Tukey’s multiple comparison test

Ngf has a critical role in differentiation and survival of neuronal populations in vivo and has similar effect on PC12 cells in culture. In the presence of Ngf, these cells undergo mitotic arrest and progressive differentiation into cholinergic neurons that develop a network of nerve fibers (Greene 1978; Greene and Tischler 1976). Ngf is sufficient to preserve the differentiated state of PC12 cells, even in the absence of serum in the growth medium. In fully differentiated PC12 cells deprived of Ngf, the neurite network quickly collapses and cells die by apoptosis (Mesner et al. 1995). This process—associated to increased expression and processing of amyloid precursors protein App, leading to overproduction of amyloid A*β* peptides—bears some resemblance to the degenerative process occurring in AD (Matrone et al. 2008b; Zhang et al. 2011). Because miR-21 enhances Ngf signaling and differentiation of PC12 cells (Figs. 3, 4, 5), we asked whether it might affect PC12 cell neuronal degeneration and death observed upon Ngf deprivation. To evaluate this possibility, PC12 cells—ectopically expressing or not miR-21—were treated with Ngf for 10 days to achieve full neuronal differentiation and then deprived of Ngf for 24, 48, and 72 h. We examined cell morphology upon staining with *α*-tubulin antibody (Fig. 6a) and investigated cell survival by cell count, trypan blue exclusion assay, and DAPI staining (Fig. 6b, c). Control cells displayed a drastic reduction of the number of nuclei and a strong retraction of neurite arborization from 24 to 72 h after Ngf deprivation. These phenomena were very attenuated in PC12 cells ectopically expressing miR-21, which presented a remarkable reduction of degeneration of the neuritic network upon deprivation of Ngf (Fig. 6a). Concomitantly, cell survival in PC12/miR-21 cells was strongly increased at 24, 48, and 72 h of Ngf deprivation (Fig. 6b, c). These assays demonstrate a protective role of microRNA-21 against cell death and neuritic degeneration in differentiated PC12 cells deprived of Ngf. Following Ngf deprivation, terminally differentiated PC12 cells show an increase in the amount of App protein (Matrone et al. 2008b). We measured App levels by immunoblotting in PC12 miR-21 and control cells—terminally differentiated by 10 days of treatment with Ngf—at different times after Ngf removal from the culture medium: 10 min, 30 min, 6 and 24 h (Fig. 7). PC12 miR-21 cells—either untreated or treated for 10 days with Ngf—had App levels slightly higher than control cells. App levels in control cells strongly increased starting at 10 min after Ngf removal. In cells ectopically expressing miR-21, instead, the amount of App slightly decreased following Ngf deprivation. These data indicate that miR-21 may restrain App production.

## Discussion

MicroRNAs are involved in fine-tuning gene expression in fundamental cellular processes (Kosik 2006). We identify a variety of microRNAs whose expression is modulated by Ngf in the course of PC12 cell differentiation. Promoters of many of them are enriched in Creb binding sites, an indication that Ngf may regulate their expression via Creb. Predicted targets of miRs modulated by Ngf are strongly enriched of MAPK and Pi3 K/Akt signaling components (Table 1). This was confirmed by target analysis of ten selected Ngf-modulated microRNAs (Table 2). Therefore, it appears that the microRNAs we have identified may participate in tuning neurotrophin signaling. Besides MAPK and Akt signaling, Ngf-modulated miRs seem to preferentially target other neuronal relevant pathways such as axon guidance and regulation of actin cytoskeleton, focal adhesion and GAP junctions, glutamergic, dopaminergic, cholinergic, and GABA-ergic synapses, long-term depression, circadian rhythms, mental disorders, and AD (Tables 1, 2). This is also evident from the analysis of single selected miRs: miR-21, miR-29c, miR-30c, miR-93, miR-103, miR-132, miR-212, miR-207, miR-691, and miR-709. MiR-21 upregulation by Ngf (Fig. 2 and Table S1) is in agreement with a previous report (Mullenbrock et al. 2012). Ngf may stimulate miR-21 expression through the AP-1 transcription factor, which is preferentially induced in PC12 cells and is known to upregulate miR-21 (Mullenbrock et al. 2012; Talotta et al. 2009). Validated targets of miR-21 include the apoptosis regulator PDCD4, the negative regulators of tyrosine kinase receptor signaling Sprouty1-2 and Pten, and the inhibitor of neurite growth Rtn4—all implicated in neurotrophin signaling. MiR-29c targets *β*-secretase 1 (Bace1)—a protein involved in App processing and mutated in familial AD (Zong et al. 2011)—and is downregulated in Huntington’s disease models (Lee et al. 2011); miR-30c is modulated by tyrosine kinase receptors and targets Bdnf mRNA (Mellios et al. 2008) as well as mRNAs encoding proteins involved in AD (App), neurotrophin signaling, and axon guidance; miR-93 has been implicated in neurogenesis and control of App protein level (Brett et al. 2008, Maes et al. 2009); miR-103 targets mRNAs encoding ID2—an inhibitor of neuronal differentiation (Annibali et al. 2012)—and neurofibromin, involved in a disease characterized by learning disabilities (Paschou et al. 2012); little is known about miR-207; miR-691 is expressed at relatively high levels in retina (Loscher et al. 2008); miR-709 is upregulated by stress in hippocampus and prefrontal cortex (Babenko et al. 2012) and it is a regulator of the injury response in the peripheral nervous system (Adilakshmi et al. 2012); mir-132 and miR-212 have been implicated in mechanisms of synaptic plasticity, drug addiction, mental diseases, and neurological disorders (Wanet et al. 2012). All this indicates that neurotrophins may affect a variety of nervous system relevant processes by controlling expression of multiple miRs.

On the basis of our results, we propose miR-21 as positive regulator of Ngf signaling. MiR-21 augments MAPK and Akt phosphorylation in response to Ngf and contributes to an increased expression of Vgf, a gene specifically induced by neurotrophins (Figs. 5 and S2). MiR-21 was shown to enhance MAPK and Akt signaling by targeting Pten and Spry2 (Sprouty 2), negative regulators of tyrosine kinase receptor signaling (Mei et al. 2012; Hatley et al. 2010; Hausott et al. 2009). Sprouty 2 and 4, in particular, negatively modulate TrkA and TrkB signaling (Alsina et al. 2012). The view of miR-21 as enhancer of Ngf signaling is consistent with its capability of stimulating PC12 cell differentiation in response to Ngf (Fig. 3), probably through TrkA receptor signaling. Coherently with this finding, miR-21 inhibition hampers neurite outgrowth of DRG neurons in response to Ngf, while ectopic expression of mR-21 seems to increase nerve fiber growth (Figs. 4 and S1). This evidence is indicative of a possible role of miR-21 on axonal development and is concordant with data showing that nerve fiber outgrowth in DRGs is enhanced by miR-21 overexpression (Strickland et al. 2011). In other cell types, instead, expression of miR-21 following activation of different tyrosine kinase receptors supports proliferation rather than differentiation (Krichevsky and Gabriely 2009). This may be explained by the different role of ERK/MAPK and Akt signaling in different cellular contexts. MAPK pathway activation in response to neurotrophins is known to promote neuronal differentiation, whereas it promotes proliferation in response to stimuli like epidermal growth factor or interleukin-6. Analogously, miR-21 knock down either enhances or suppresses cancer cell growth, according to cell type (Papagiannakopoulos et al. 2008; Wang et al. 2009).

Since Ngf is crucial for survival and nerve fiber arborization in PC12 cells, an enhancement of Ngf signaling by miR-21 may as well explain its anti-neurodegenerative properties, the most striking of our results (Figs. 6, 7). A defect of neurotrophin signaling has been involved in AD and other neurodegenerative diseases. Such a neuroprotective role of miR-21 is coherent with recent evidence: miR-21 protects neurons from ischemic death (Buller et al. 2010), it is induced by axotomy and promotes axon growth (Strickland et al. 2011), regulates the response of astrocytes following spinal cord injury (Bhalala et al. 2012) in microglia, targets the apoptotic inducer Fas ligand (FasL), and has a neuroprotective role (Zhang et al. 2012).

In conclusion, our findings identify an additional mechanism for sustaining neurotrophin signaling and indicate that altered regulation of miR-21 and possibly other neurotrophin-modulated microRNAs may contribute to neurodegeneration.

## Materials and Methods

### Cell Culture

PC12 cells were cultured in RPMI medium containing 10 % horse serum (HS) and 5 % fetal bovine serum (FBS) in a CO_2_ humidified incubator at 37 °C. Mouse salivary gland ß-Ngf was kindly provided by Bioway Company, China. Lyofilized, sterile samples of 17 μg each were dissolved in 1 ml sterile 0.9 % NaCl and kept at 4 °C until use. Ngf was used at a concentration of 100 ng/ml. Differentiation was achieved by treating cells with Ngf for 10 days, as described (Matrone et al. 2008b). In the Ngf deprivation experiments, cells were washed three times with PBS, once with serum-free medium, and placed in serum-free media without Ngf (Matrone et al. 2008b).

Dorsal root ganglia (DRGs) were collected from Wistar rats at P0 (postnatal day 0) to P5 in PBS, digested with 0.02 % trypsin for 30 min at 37 °C, and further dissociated with a Pasteur pipette. Approximately 100,000/200,000 cells were plated on slides coated with poly-d-lysine and cultured in DMEM F12 plus 10 % FBS serum without Ngf.

### Expression Plasmids

To obtain the miR-21 expressing lentiviral vector pRRL-21, a region of about 300 bp containing the miR-21 DNA sequence was amplified from Jurkat cells genomic DNA with cgcgcgcggaccggatcaaatcctgcctgactgt (forward) and cgcgcgcgtagctgccaccagacagaaggacc (reverse) primers and cloned into RsrI and NheI sites of the lentiviral vector #1074.1071.hPGK.GFP.WPRE.mhCMV.dNgfR.SV40PA, kindly provided by Prof Luigi Naldini (TIGET, Milan). The pRRL-control vector was obtained by substituting the miR-21 precursor fragment (RsrI–NheI) with a sequence encoding a hairpin yielding a 22-mer RNA designed to lack homology to any human gene. The miR-21 sponge expression plasmid CMV-d2eGFP-21 was purchased by Addgene (plasmid #21972); it incorporates several miR-21 binding sequences in the 3’UTR of a destabilized EGFP gene (Ebert et al. 2007). The miR-21 sponge lentiviral plasmid was obtained by replacing the miR-233 target sequence with the miR-21 sponge, in the lentiviral vector described in Gentner et al. 2009.

### Cell Transfection and Lentivirus Infection

PC12 cells were plated on poly-d-lysine and transfected with plasmid DNA by using Lipofectamine LTX plus (Invitrogen) following the manufacturer’s instructions. 24 h later, cells were stimulated with Ngf in RPMI medium containing 5 % HS and 2,5 % FBS. After 48 h, cells were fixed with 4 % paraformaldehyde and examined by a Nikon Eclipse TE2000-E microscope. DRGs were dissected and plated on poly-d-lysine in the absence of Ngf; the day after, DRGs were transfected with plasmid CMV-d2eGFP-21 and plasmid encoding EGFP as control by means of lipofectamine LTX plus (Invitrogen), following the manufacturer’s instructions. After 4 h, cells were stimulated with 100 ng/ml Ngf for 12 h. Neurite length in PC12 cells and DRG neurons was evaluated by Fiji software (http://fiji.sc/Fiji). Lentivirus was prepared and cell infections were performed as described (Annibali et al. 2012).

### Northern Blotting

Northern blotting was carried out as described (Annibali et al. 2012). Total RNA was extracted with TRIzol Reagent (Life Technologies) according to manufacturer’s instructions, fractionated by 15 % poly-acrylamide gel electrophoresis in 1X TBE, 7M urea and transferred onto Amersham Hybond-NX nylon membrane (GE Healthcare). Membranes were hybridized to 32^P^-labeled DNA oligonucleotides complementary to the sequence of mature miR-21 TCAACATCAGTCTGATAAGCTA and U2 small nucleolar RNA (snoRNA) GCAGGGGCCATGCTAATCTTCTCTGTATCG, used to normalize expression levels.

### Real-Time PCR

Quantitative real-time PCR (RT-PCR) was performed using the miScript SYBR Green PCR Kit in 96-well plates according to the manufacturer’s instructions. Each 20 μl reaction contained 2.5 ng template cDNA, 2 μl universal primer, and 2 μl miR specific primer (from Qiagen) or U6 snoRNA primer as control. Real-time PCR reactions were performed by an Applied Biosystem 7500 apparatus. Expression data were calculated by 7500 software v2.0.5 (Applied Biosystems) according to the ∆∆Ct method and expressed as relative quantities after normalization to U6 level.

### Microarray Analysis

Before proceeding with microarray profiling, the efficacy of Ngf was checked by RT-PCR expression analysis of PC3, a known Ngf immediate-early responsive gene (data not shown). RNA was extracted with TRIzol and 5 μg RNA from each sample were labeled with Hy5™ and Hy3™ fluorescent labels using the miRCURY™ LNA Array power labeling kit (Exiqon, Denmark), according to the procedure described by the manufacturer. Hy5™-labeled samples and Hy3™-labeled reference RNA sample were mixed pair-wise and hybridized to the miRCURY™ LNA Array version 11.0 (Exiqon, Denmark), which contains capture probes targeting 514 unique miRs from human, mouse, and rat registered in miRBASE version 12.0, and spike-in controls. The hybridization was carried out as recommended by the vendor. After hybridization, the microarray slides were scanned using a Perkin-Elmer Scan Array and images analyzed by the GenePix Pro 6.0 program. Analysis of the scanned slides indicated successful labeling of RNAs as evidenced by the signal intensities of all capture probes for the control spike-in oligonucleotides. For each slide, spots with signal to background ratio below 1.5 and flagged spots were filtered out. Systematic bias in the data was removed by applying the LOWESS normalization. To establish the significance of observed regulation for each miR, Student’s *t* test was used and the *p* values were corrected for multiple comparisons. Finally, miRs with a *p* value less than 0.05 and a FC greater than 1.7 or less than −1.7 (FC at least 1.7 in absolute value) were considered. Heat maps were generated by two-way hierarchical clustering. Microarray data are deposited in GEO and accessible at the record # GSE46827.

### Bioinformatics Analysis

TargetScan (6.2; http://targetscan.org/) and DIANA microT (http://diana.cslab.ece.ntua.gr/microT/) software was used to predict microRNA targets. Targets were investigated through DIANA mir-Path (http://diana.cslab.ece.ntua.gr/) and KEGG Pathway, containing over 20,900 annotated human genes (www.genome.jp/kegg/pathway.html). Creb binding sites were investigated via JASPAR (http://jaspar.genereg.net/).

### Immunoblotting and Immunohistochemistry

Whole-cell protein extracts were prepared from cells lysed in RIPA buffer. Samples were separated through SDS PAGE gels, transferred to Hybond ECL membranes (GE Healthcare), and treated with appropriate antibodies. Staining was performed by the SuperSignal Chemiluminescent Substrate from Pierce. ImageJ software (http://rsbweb.nih.gov/ij/) was used for densitometric analysis; western blot quantification was normalized against loading controls. Immunohistochemical detection was performed as described (Annibali et al. 2012).

Antibodies used: phospho-Akt(Ser473) and phospho-p44/42 MAPK (ERK1/2) (Thr202/Tyr204) antibodies were from Cell Signaling, Vgf antibody [24] was kindly provided by A. Levi, monoclonal anti-*β*-Actin–peroxidase clone Ac-15 (A3854) and anti-*α*-Tubulin (T6199) were from Sigma, beta III tubulin antibody was from Abcam, and mouse monoclonal anti-App (22C11) from Millipore. Horseradish peroxidase secondary antibodies were from Chemicon and Protein A peroxidase from Sigma.

### Statistical Analysis

Values are expressed as mean ± s.e.m. Statistical analyses were performed by Student’s *t* test, hypergeometric function, and ANOVA, followed by the Newman–Keuls or Tukey’s multiple comparison tests. Statistical significance was accepted at the 95 % confidence level (*p* value ≤0.05).

## Electronic supplementary material

Below is the link to the electronic supplementary material.

Supplementary material 1 (DOCX 364 kb)
