# Supplementary material for: MiR-21 is an Ngf-Modulated MicroRNA That Supports Ngf Signaling and Regulates Neuronal Degeneration in PC12 Cells
Source: Neuromolecular Med. 2014 Feb 4;16(2):415–30. doi: 10.1007/s12017-014-8292-z (PMC4019824; doi:10.1007/s12017-014-8292-z)
Supplement: Supplementary file 1 — Supplementary material 1 (DOCX 364 kb) [file 12017_2014_8292_MOESM1_ESM.docx]

**Supplementary material**

**Table S1. Ngf regulates the expression of a variety of microRNAs in PC12 cells**

MicroRNAs differentially expressed in PC12 cells treated with Ngf for 1h (A), 3h (B), 6h (C), 24 h (D), and 10 days (E), with reference to untreated PC12 cells. MiRs are ranked according to decreasing Fold Change (FC) values (negative values indicate downregulation). Only miRs satisfying the two threshold criteria – FC ≤-1.7 or ≥ 1.7; p value ≤0.05 in two-tailed Student's t-test – are reported.

**Table S1** Differentially expressed microRNAs

| **A.** 1 h Ngf treatment | FC | p-value |
| --- | --- | --- |
| mmu-miR-709 | 2.0 | 0.03 |
| hsa-miR-665 | 2.0 | 0.05 |
| hsa-miR-299-3p | 1.9 | 0.03 |
| hsa-miR-425-3p/mmu-miR-425-3p | 1.8 | 0.02 |
| mmu-mir-1195 | 1.8 | 0.04 |
| hsa-miR-1827 | 1.7 | 0.02 |
| hsa-miR-193a-3p/mmu-miR-193a-3p/rno-miR-193-3p | -1.8 | 0.01 |
| hsa-miR-130a-3p/mmu-miR-130a-3p/rno-miR-130a-3p | -1.8 | 0.02 |
| hsa-miR-320b | -1.9 | 0.02 |
| miR-29c (hsa-miR-29c-3p/mmu-miR-29c-3p/rno-miR-29c-3p) | -2.0 | 0.01 |
| hsa-miR-615-5p/mmu-miR-615-5p | -2.1 | 0.02 |
| hsa-miR-33a-5p | -3.1 | 0.03 |

| **B.** 3 h Ngf treatment | FC | p-value |
| --- | --- | --- |
| mmu-miR-582-3p | 2.0 | 0.04 |
| hsa-miR-1285-3p | 2.0 | <0.01 |
| mmu-miR-691 | 1.9 | 0.01 |
| hsa-miR-299-3p | 1.8 | 0.04 |
| hsa-miR-665 | 1.8 | 0.07 |
| mmu-mir-1195 | 1.7 | 0.05 |
| miR-93 (hsa-miR-93-5p/mmu-miR-93-5p/rno-miR-93-5p) | -1.8 | 0.05 |
| mmu-miR-33-5p/rno-miR-33-5p | -1.7 | 0.01 |
| miR-29c (hsa-miR-29c-3p/mmu-miR-29c-3p/rno-miR-29c-3p) | -1.8 | 0.02 |
| hsa-miR-33a-5p | -2.6 | 0.02 |

| **C.** 6 h Ngf treatment | FC | p-value |
| --- | --- | --- |
| miR-21 (hsa-miR-21-5p/mmu-miR-21-5p/rno-miR-21-5p) | 2.9 | <0.01 |
| hsa-miR-938 | 2.1 | 0.05 |
| hsa-miR-1264 | 1.9 | 0.01 |
| mmu-miR-1196-5p | 1.9 | <0.01 |
| hsa-miR-605 | 1.8 | 0.03 |
| mmu-miR-207/rno-miR-207 | 1.8 | 0.02 |
| hsa-miR-99b-3p/mmu-miR-99b-3p/rno-miR-99b-3p | 1.8 | 0.02 |
| hsa-miR-1260a | 1.8 | <0.01 |
| mmu-miR-33-5p/rno-miR-33-5p | 1.8 | 0.01 |
| hsa-miR-767-5p | 1.7 | 0.04 |
| hsa-miR-597 | 1.7 | 0.00 |
| hsa-miR-551b-5p | 1.7 | 0.04 |
| mmu-miR-667-3p | 1.7 | 0.01 |
| hsa-miR-519d | 1.7 | 0.01 |
| hsa-miR-1248 | -2.2 | 0.02 |

| **D.** 24 h Ngf treatment | FC | p-value |
| --- | --- | --- |
| hsa-miR-193a-3p/mmu-miR-193a-3p/rno-miR-193-3p | -3.1 | 0.03 |
| hsa-miR-1275 | -2.3 | 0.01 |
| hsa-miR-98-5p/mmu-miR-98-5p/rno-miR-98-5p | -2.2 | 0.01 |
| hsa-miR-331-3p/mmu-miR-331-3p/rno-miR-331-3p | -2.4 | 0.04 |
| hsa-miR-665 | -1.9 | 0.03 |
| mmu-miR-1186a | -1.9 | 0.04 |
| mmu-miR-711 | -1.8 | 0.02 |
| mmu-miR-466a-5p | -1.8 | 0.02 |
| mmu-miR-465c-5p | -1.8 | 0.02 |
| hsa-miR-375/mmu-miR-375/rno-miR-375-3p | -1.7 | 0.04 |
| hsa-miR-513a-3p | 1.7 | 0.04 |
| hsa-miR-138-3p/mmu-miR-138-3p/rno-miR-138-3p | 1.7 | 0.05 |
| hsa-miR-766-3p | 1.7 | 0.01 |
| hsa-miR-518a-5p/ hsa-miR-527 | 1.8 | 0.05 |
| hsa-miR-584-5p | 2.0 | 0.04 |
| hsa-miR-519d | 2.1 | 0.05 |
| hsa-miR-335-3p/mmu-miR-335-3p | 2.4 | 0.01 |

| **E.** 240 h Ngf treatment | FC | p-value |
| --- | --- | --- |
| hsa-miR-638 | 7.5 | 0.02 |
| hsa-miR-557 | 3.8 | 0.01 |
| miR-21 (hsa-miR-21-5p/mmu-miR-21-5p/rno-miR-21-5p) | 3.2 | 0.02 |
| hsa-miR-202-3p | 3.0 | 0.01 |
| hsa-miR-518a-5p/ hsa-miR-527 | 2.9 | 0.05 |
| hsa-miR-138-5p/mmu-miR-138-5p/rno-miR-138-5p | 2.9 | 0.03 |
| hsa-miR-675-5p | 2.9 | 0.04 |
| hsa-miR-938 | 2.7 | 0.05 |
| mmu-miR-290-5p/rno-miR-290 | 2.6 | 0.02 |
| hsa-miR-1264 | 2.5 | 0.02 |
| hsa-miR-629-3p | 2.5 | 0.05 |
| hsa-miR-302c-5p | 2.5 | 0.02 |
| hsa-miR-551b-5p | 2.5 | 0.05 |
| hsa-miR-1260a | 2.4 | 0.04 |
| hsa-miR-519d | 2.4 | 0.02 |
| hsa-miR-605 | 2.4 | 0.02 |
| hsa-miR-554a | 2.3 | 0.04 |
| hsa-miR-597 | 2.2 | 0.04 |
| hsa-miR-22-3p/mmu-miR-22-3p/rno-miR-22-3p | 2.1 | 0.03 |
| hsa-miR-767-5p | 2.1 | <0.01 |
| hsa-miR-1290 | 2.1 | 0.01 |
| hsa-miR-887 | 2.0 | <0.01 |
| mmu-miR-207/rno-miR-207 | 2.0 | 0.02 |
| hsa-miR-520d-5p | 2.0 | 0.01 |
| hsa-miR-874/mmu-miR-874-3p/rno-miR-874-3p | 1.9 | 0.05 |
| hsa-miR-494/mmu-miR-494-3p/rno-miR-494-3p | 1.9 | 0.01 |
| hsa-miR-129-3p | 1.9 | 0.05 |
| mmu-miR-675-5p | 1.8 | 0.02 |
| mmu-miR-503-3p | 1.8 | 0.01 |
| hsa-miR-933 | 1.8 | 0.03 |
| mmu-miR-804 | 1.8 | 0.01 |
| hsa-miR-1275 | 1.8 | 0.02 |
| hsa-miR-183-3p/mmu miR-183-3p | 1.8 | 0.04 |
| hsa-miR-203a/mmu-miR-203-3p/rno-miR-203a-3p | 1.7 | 0.04 |
| hsa-miR-516a-5p | 1.7 | 0.02 |
| hsa-miR-378a-5p/mmu-miR-378a-5p/rno-miR-378a-5p | -1.7 | 0.05 |
| hsa-miR-16-5p/mmu-miR-16-5p/rno-miR-16-5p | -1.7 | 0.05 |
| hsa-miR-342-3p/mmu-miR-342-3p /rno-miR-342-3p | -1.7 | 0.03 |
| hsa-miR-195-5p/mmu-miR-195a-5p /rno-miR-195-5p | -1.7 | 0.05 |
| mmu-miR-1195 | -1.8 | 0.04 |
| hsa-miR-106a-5p | -1.8 | 0.02 |
| hsa-miR-106b-5p/mmu-miR-106b-5p/rno-miR-106b-5p | -1.8 | <0.01 |
| hsa-miR-30b-5p/mmu-miR-30b-5p/rno-miR-30b-5p | -1.9 | 0.05 |
| hsa-miR-30e-5p/mmu-miR-30e-5p/rno-miR-30e-5p | -1.9 | 0.05 |
| miR-30c (hsa-miR-30c-5p/mmu-miR-30c-5p/rno-miR-30c-5p) | -1.9 | 0.02 |
| hsa-miR-363-5p/rno-miR-363-5p | -2.0 | 0.01 |
| hsa-miR-301-3p/mmu-miR-301-3p/rno-miR-301-3p | -2.0 | 0.03 |
| hsa-miR-15b-5p/mmu-miR-15b-5p/rno-miR-15b-5p | -2.1 | 0.02 |
| hsa-miR-150-5p/mmu-miR-150-5p/rno-miR-150-5p | -2.2 | 0.02 |
| rno-miR-466c-5p | -2.3 | <0.01 |
| miR-93 (hsa-miR-93-5p/mmu-miR-93-5p/rno-miR-93-5p) | -2.3 | 0.03 |
| hsa-miR-25-3p/mmu-miR-25-3p /rno-miR-25-3p | -2.3 | 0.01 |
| hsa-miR-665 | -2.4 | 0.02 |
| mmu-miR-691 | -2.7 | <0.01 |
| hsa-miR-190a/mmu-miR-190a-5p /rno-miR-190a-5p | -2.7 | 0.01 |
| hsa-miR-668/mmu-miR-668-3p | -2.9 | 0.05 |
| hsa-miR-210/mmu-miR-210-3p /rno-miR-210-3p | -3.0 | 0.01 |
| hsa-miR-1248 | -3.9 | 0.01 |

**Figure S1**

**
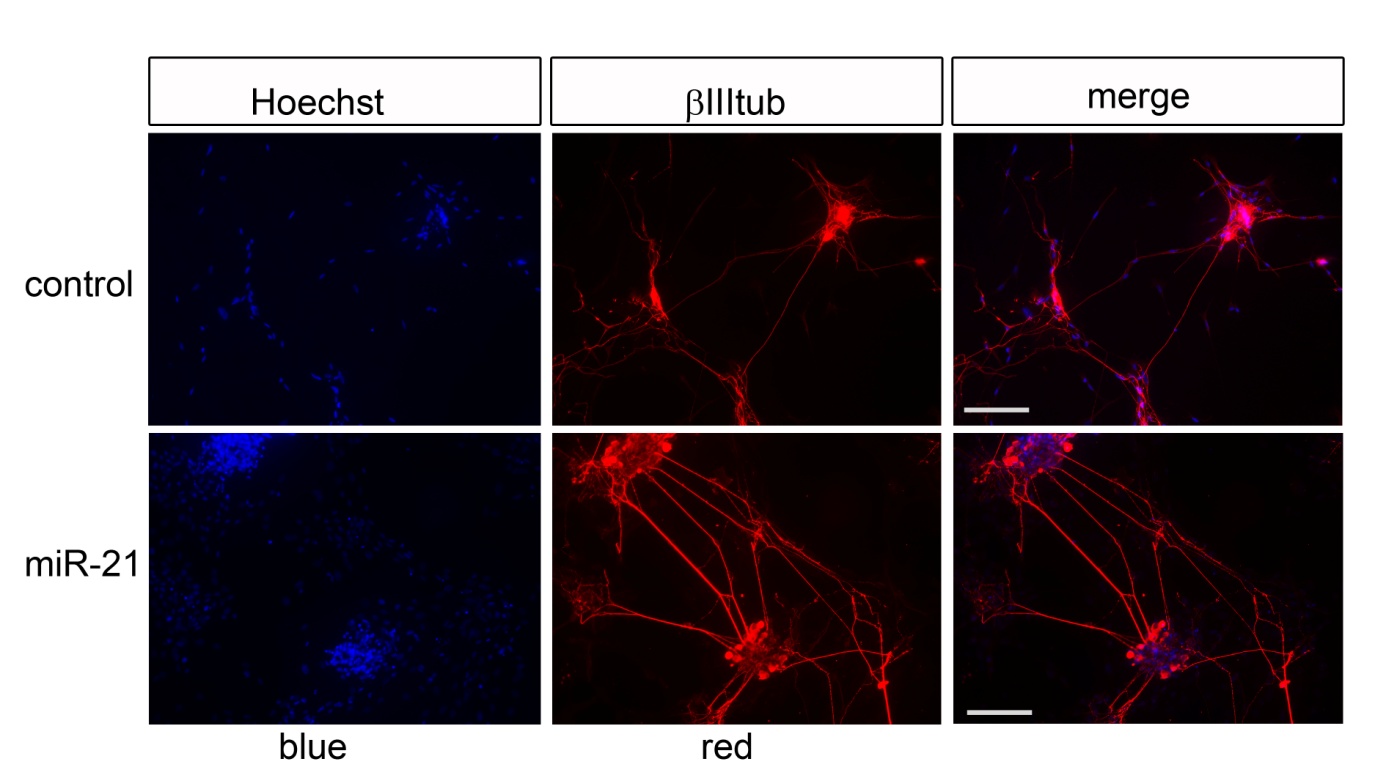
**

**Figure S2**

**Figure S1**

**MiR-21 stimulates axonal growth in DRG neurons.** Immunofluorescence staining of ß-III-tubulin in control and miR-21 overexpressing DRG neurons. Nerve fibers in miR-21 neurons appear bigger than in control neurons.

**Figure S2**

**MiR-21 enhances Akt and MapK phosphorylation.** Immunoblotting of Akt, phospho-Akt (Ser 473), MapK, and phospho-p42/44 MapK (Thr 202/Tyr 204) - at the indicated times of NGF treatment - in control and miR-21 ectopically expressing PC12 cells (left panel), and in PC12 cells infected with a lentivirus encoding miR-21 sponge and control (right panel).
